# Formation of Die Soldering and the Influence of Alloying Elements on the Intermetallic Interface

**DOI:** 10.3390/ma14071580

**Published:** 2021-03-24

**Authors:** Marius Kohlhepp, Peter J. Uggowitzer, Marc Hummel, Heinz Werner Höppel

**Affiliations:** 1 Department of Materials Science & Engineering, Institute I: General Materials Properties, Friedrich-Alexander-Universität Erlangen-Nürnberg, 91058 Erlangen, Germany; hwe.hoeppel@fau.de; 2 Laboratory of Metal Physics and Technology, Department of Materials, ETH Zurich, 8093 Zurich, Switzerland; peter.uggowitzer@mat.ethz.ch; 3 Chair of Nonferrous Metallurgy, Montanuniversität Leoben, A-8700 Leoben, Austria; 4 AUDI AG, Auto-Union-Straße 1, 85057 Ingolstadt, Germany; marc.hummel@audi.de

**Keywords:** aluminum, die soldering, intermetallic phases, high-pressure die casting, chromium, manganese, molybdenum, cobalt

## Abstract

Die soldering of die castings is a serious problem in the aluminum casting industry. The precise mechanism, the influence of the alloy composition, and the options for prevention have not yet been fully elaborated. A well-established solution for alloys with low iron content is the addition of manganese. However, up to 0.8 wt.% is necessary, which increases the amount of brittle phases in the material and consequently reduces ductility. Immersion tests with 1.2343 tool steel and pure aluminum as well as a hypoeutectic AlSi-alloy with Mn, Mo, Co, and Cr additions were carried out to systematically investigate the formation of die soldering. Three different intermetallic layers and a scattered granular intermetallic phase formed at the interface between steel and Al-alloy after immersion into the melt for a duration of 6 min at 710 °C. The combined presence of the irregular, needle-shaped β-Al_5_FeSi phase and the surrounding alloy was responsible for the bond between the two components. Mn and Mo inhibited the formation of the β-phase, and instead promoted the α_C_-Al_15_(Fe,X)_3_Si_2_ phase. This led to an evenly running boundary to the AlSi-alloy and thus prevented bonding. Cr has proven to be the most efficient addition against die soldering, with 0.2 wt.% being sufficient. Contrary to the other elements investigated, Cr also reduced the thickness of the intermetallic interface.

## 1. Introduction

**Figure 1:**
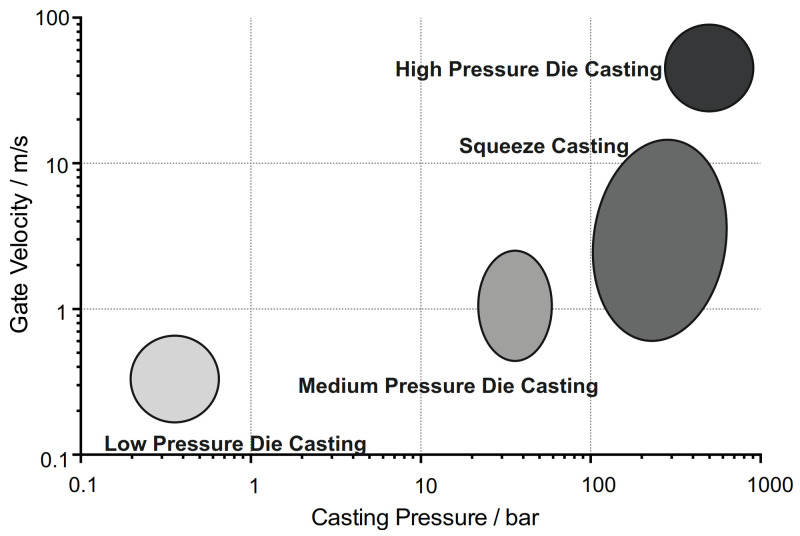
Comparisons of casting pressures to gate velocities for numerous die-casting processes (data from [2]).

High-pressure die casting (HPDC) is one of the most common methods for mass production of aluminum alloy parts. Because of the low cycle time of the casting operation, as well as the good mechanical properties and castability, HPDC possesses a high productivity and operational cost efficiency [1]. This makes it popular in various fields of the global industry. However, the high velocity of the melt injection and the great pressure applied after the filling, as illustrated in Figure 1, creates harsh conditions for the die material. As a result, die soldering is one of the main die failure modes, leading to malfunctioning of die inserts, production downtimes, and damage to the casting components. This phenomenon occurs when the liquid aluminum melt reacts with the die to form a solid compound, which prevents faultless demolding.

Most studies agree that a combination of erosion and intermetallic build-up must take place for die soldering to occur. Chu et al. [3] showed that the high speed of the melt leads to washout of the protective film (coating or lubricant) on the die surface. At these areas, the aluminum melt comes into direct contact with the die steel, which makes metallurgical reaction and build-up of intermetallic phases possible. The main reason for the formation of intermetallic phases at the interface is the strong affinity of aluminum and iron, which leads to strong interdiffusion on contact [4]. Because the intermetallic phases found in HPDC samples are similar to those in immersion experiments and diffusion couples, most research on die soldering has been carried out with these methods.

Shankar and Apelian [5,6,7], who published several studies about the phenomenon, examined the reaction for a hypoeutectic AlSi-alloy. For them, as for Nazari and Shabestari [8], the diffusion of iron into the melt proved to be the critical factor, responsible for the thickness of the intermetallic layer. Shankar and Apelian divided the formation of the intermetallic buildup into different steps, starting with the erosion of softer regions and the formation of hemispherical pits [7]. Next, binary phases form in these pits, which subsequently react with the melt to form ternary AlFeSi-phases. These phases grow pyramidal shaped from the pits into the aluminum alloy, creating the metallurgical bond and thus die soldering. Han and Viswanathan [9], on the other hand, focused their research on the transportation of aluminum into the steel. They split the steel surface into different regions, depending on the aluminum concentration, and therefore the corresponding intermetallic phase. For die soldering to occur, a part of the interface has to become liquid. Han and Viswanathan defined that temperature as the critical temperature T_C_. When die soldering takes place, the surface temperature of the die T_D_ must therefore be higher than T_C_.

Besides the temperature, various other factors have a major influence on the intermetallic layer at the interface. Although die soldering has been the subject of many studies in recent years [3,5,6,7,8,9,10,11,12,13,14,15], the formation mechanism and the influence of the alloy composition in particular are not yet fully understood. In the present work, a series of immersion experiments were conducted to systematically analyze the structure and morphology of the intermetallic layers. The aim of the study is to determine the precise mechanism of die soldering and how it is affected by different alloy compositions. For this purpose, a reference Al-Si cast alloy was systematically modified by addition of manganese, molybdenum, cobalt, and chromium in order to be able to evaluate differences in the occurrence of the intermetallic interfaces, and to derive measures thereof to prevent die soldering.

## 2. Materials and Methods

**Figure 2:**
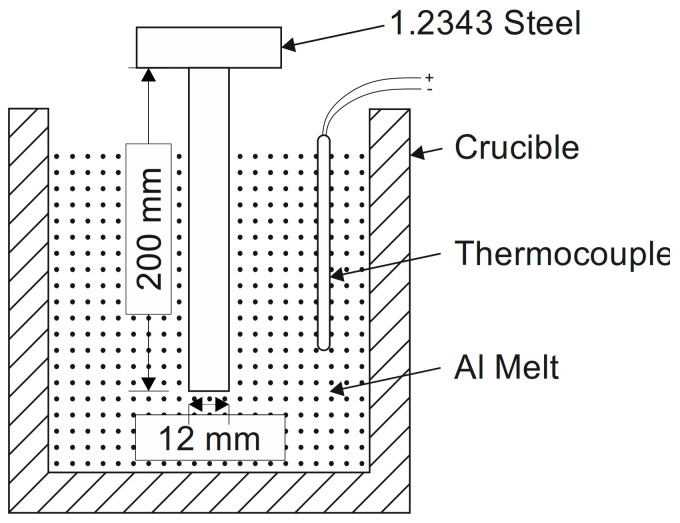
Schematic of the setup used for the dipping experiments.

To investigate the intermetallic layers at the interface between the die and the alloy, dipping experiments were carried out. The experimental set up is illustrated in Figure 2. Uncoated ejector pins of 1.2343 hot work steel with a 12 mm diameter and 200 mm length were used, with a chemical composition comparable to the common H13 steel (Table 1). The pins were hardened and tempered to achieve a Rockwell hardness of HRC 45–48 and a surface finish similar to a commercial die.

For each experimental run, 3 kg of aluminum alloy was melted in a graphite crucible. The chemical composition of the reference alloy (Table 1) was measured by spark spectroscopy. The melt temperature was maintained at 710 °C and controlled using a temperature controller via a K-type thermocouple. The ejector pins were preheated to 300 °C before dipping them into the aluminum melt for 360 s. Afterward, the pins were cooled to room temperature in still air. Furthermore, three different durations were used for the pure aluminum dipping experiments to investigate the growth rate of the intermetallic interface.

Various chemical elements (Mn, Mo, Co, Cr) were added in three stages to the reference alloy (Table 1) in order to investigate their respective influence on the intermetallic layers at the interface (Table 2). After immersion, the samples were taken out of the melt and cooled in air. For microstructural studies, they were sectioned at four positions for better statistics, then mounted, ground, and polished. Deep etching was conducted with NaOH solution for 5 min at 50 °C. Optical microscopy was used to investigate the morphology of the intermetallic layers. The intermetallic phases were analyzed using a field emission scanning electron microscope (ZEISS SIGMA VP, Oberkochen, Germany) in combination with an Oxford Instruments X-Max detector (Oxford Instruments plc., Abingdon, UK). The backscatter electron detector was used to discern between the layers. The phases and layers were identified by energy-dispersive X-ray spectroscopy (EDS) and by comparison with data in literature. To investigate the effect of the alloying elements, EDS mappings were carried out.

## 3. Results and Discussion

### 3.1. Mechanism of Die Soldering

#### 3.1.1. Tool Steel and Pure Aluminum

**Figure 3:**
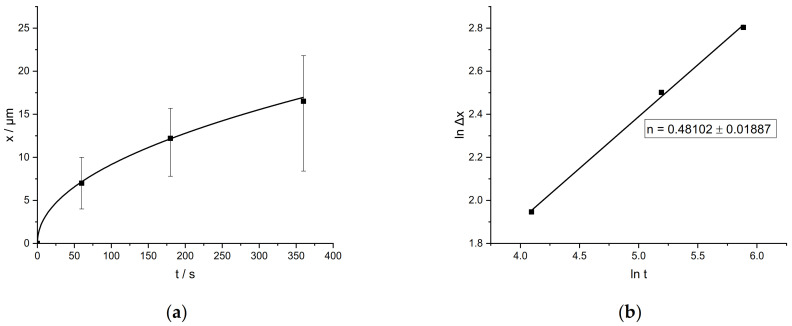
(**a**) Plot of intermetallic layer thickness vs. immersion time; (**b**) plot of ln ∆x vs. ln t with value of n determined by linear regression using Equation (1).

Figure 3a shows the evolution of the intermetallic layer thickness at the interface after immersion of the 1.2343 tool steel into the pure aluminum melt at different dipping times. As shown, the layer thickness increased according to a parabolic function. Various studies have indicated that the formation of the intermetallic layers is a combined process of diffusion and chemical reaction at the reaction front [6,13,16].

**Figure 4:**
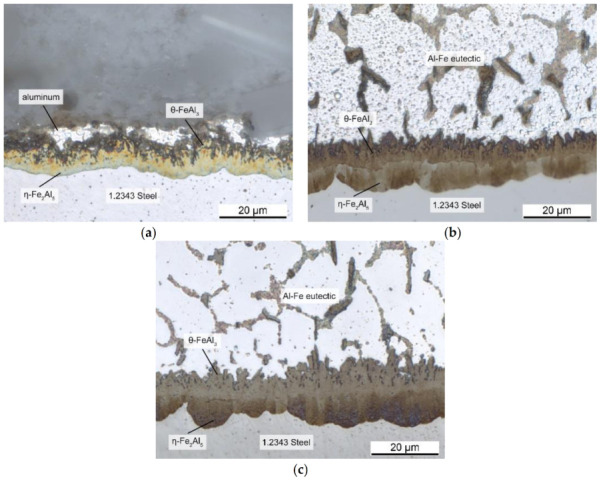
Intermetallic layers at the interface between aluminum and 1.2343 tool steel after immersion for (**a**) 60 s; (**b**) 180 s; (**c**) 360 s.

The relation between the intermetallic layer thickness ∆x and the reaction time at a given temperature can be described by the empirical relationship:

Δx = k × t^n^ (1)

where k is the parabolic rate constant and n is the kinetic exponent [17]. Linear growth implies that the reaction rate is the decisive factor, controlling the growth rate [18,19,20]. Parabolic growth, on the other hand, indicates that predominantly diffusion controls the growth. Figure 3b shows the plot of ln ∆x versus ln t for the intermetallic interface shown in Figure 4, in which linear regression analysis was used to determine a best-fit straight line. This resulted in a value of 0.48 for the kinetic exponent n. A value of 0.5 indicates a parabolic and thus a diffusion-controlled growth, whereas a value of 1.0 reflects a reaction-rate-controlled growth [18]. For longer time periods, the simultaneous dissolution of the intermetallic layers must be considered, so that more complex approaches such as Dybkov’s model [21] are necessary. However, for the timespans used in this study, the parabolic growth represents a good approximation, confirming diffusion-controlled growth of the intermetallic interface.

**Figure 5:**
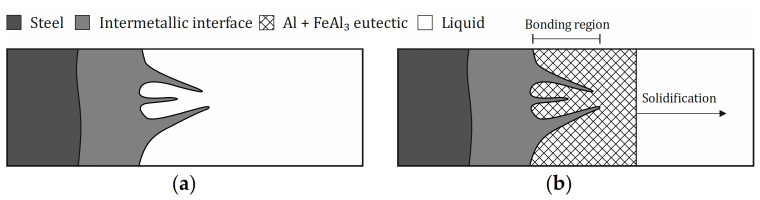
Schematic illustration for the mechanism of die soldering: (**a**) beginning of casting cycle before solidification starts; (**b**) during solidification of the Al-casting, where a connection between the die and the alloy has already formed in the bonding region.

At the interface, two compact layers could be differentiated: an Fe-rich layer consisting of η-Fe_2_Al_5_, and a θ-FeAl_3_ phase adjacent to the aluminum alloy. While the η-Fe_2_Al_5_ layer grew in a toothlike morphology into the steel, the θ-FeAl_3_ layer grew out into the aluminum alloy. Because of its irregular, needlelike shape, it formed a considerable surface area. It is important to note that a eutectic region (aluminum + FeAl_3_) formed the transition between the irregular θ-FeAl_3_ surface and the Al-alloy (in the present case, pure Al). As shown in Figure 5, this rough interface created a bond, thus initiating die soldering and impeding demolding. In the binary Al–Fe phase diagram, the solidus temperature of the aluminum–iron eutectic was 655 °C [22]. Since the HPDC operating temperature of the melt is usually around 700 °C, the eutectic region should become liquid on contact with the melt. That implies that the area around the irregularly shaped interface became liquid in every casting cycle. Since diffusion still took place, the interface layers continued to grow, which led to an increasing strengthening of the die-soldering bond. This corresponded very well with common experience from the casting industry, where die soldering gets worse over time.

Han and Viswanathan observed a similar interface structure in their studies [9]. They defined the combination of a θ-FeAl_3_ layer with an aluminum–iron eutectic responsible for die soldering. Since no soldering occurred below the eutectic temperature of 655 °C, they also concluded that both layers have to be present for die soldering. Therefore, the temperature at the interface must be higher than 655 °C, which supports the results of this study. They stated this was the critical temperature T_C_, above which the eutectic becomes liquid. The amount of liquid depends on the aluminum concentration at the interface.

#### 3.1.2. Tool Steel and AlSi-Alloy

**Figure 6:**
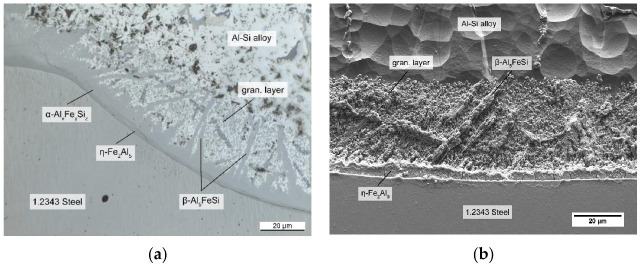
Intermetallic layers at the interface between an AlSi-alloy and 1.2343 tool steel: (**a**) optical microscope image; (**b**) SEM image after deep etching.

However, the shape and composition of the intermetallic layers changed when an AlSi-alloy was used instead of pure Al (see also [10]). Figure 6 shows the interface of the tool steel and the AlSi7-alloy after immersion. The η-Fe_2_Al_5_ layer was still present, but its shape was smoothened, and the toothlike outline had disappeared. According to Yin et al. [23], this change in morphology might be due to the silicon atoms occupying vacancies necessary for the Nabarro–Herring creep mechanism, which is responsible for the specific morphology by creating an atomic flow inside the η grain, as reported by Takata et al. [24]. The stress introduced by the transformation of α-Fe into η phase is responsible for this flow. In addition, the differences in the atomic volume might also account for the change in morphology. The θ-FeAl_3_ layer, which formed in the case of pure Al, could not be observed in the ternary system, and instead, ternary layers developed adjacent to the binary η-Fe_2_Al_5_.

The results of the EDS analyses shown in Table 3 indicated that the two layers consisted of hexagonal α_H_-Al_12_Fe_3_Si_2_ and β-Al_5_FeSi. The Fe content decreased in the order of the layers, which indicated they were built by the reaction of each layer with the melt, as noted by Shankar and Apelian [6]. The β-Al_5_FeSi phase grew in form of needles into the aluminum alloy. An eutectic surrounding the needles was still present in the ternary case, although it consisted of an AlSi-eutectic, as well as primary and eutectic solidified β-Al_5_FeSi. Furthermore a large amount of scattered granular intermetallic phase identified as cubic α_C_-Al_15_(Fe,X)_3_Si_2_ (in the following denoted as granular phase) surrounded by fcc-Al phase formed adjacent to the intermetallic layers at the interface. As noted by Cheng et al. [25], the scattered granular phase arises while the melt surrounding the intermetallic interface is still liquid. Cr, which is present in the tool steel and diffuses out of it, saturates the α_H_-Al_12_Fe_3_Si_2_ phase at the interface and enhances the transformation to the cubic α_C_-Al_15_(Fe,X)_3_Si_2_ phase. Because of the transformation α_H_ -> α_C_ + L the formed α_C_-Al_15_(Fe,X)_3_Si_2_ phase would then spall into the AlSi-alloy, which results in the particles floating above the α_H_-Al_12_Fe_3_Si_2_ layer. Mn and Cr can mostly be found in these granular phases, which is consistent with the established observations, that Cr and Mn stabilize a cubic structure and possess a higher solubility in α_C_-Al_15_(Fe,X)_3_Si_2_ [25,26]. These elements share a similar behavior by occupying iron lattice sites X. Both are only minor impurities in the alloy, with their higher concentration at the interface resulting from the diffusion out of the steel.

By taking the results of the binary system into account, it can be concluded that the combination of the irregular, needlelike interface of the β-Al_5_FeSi phase and the surrounding alloy was responsible for the metallurgical bond. The role of the granular phase regarding die soldering in a HPDC process is not fully understood, and should be considered for further research. However, the AlSi7-alloy, especially the eutectic components and the fcc-Al phase between the granular particles, had a solidus temperature lower than the casting temperature. Hence, if the die surface exceeded that temperature, the region remelted, and a liquid phase was present. On solidification, the two phases formed an interlocked structure, linking the aluminum casting to the steel.

Other studies observed a similar change of the interface when examining immersion tests with an AlSi-alloy, supporting these results. The formation of a pyramidal shaped phase reported by Shankar and Apelian [7] corresponded very well with early states of the needles observed in this study. The observations made by Winkelman et al. [26], who subdivided the corresponding region into a solid compact layer, broken layer, and a fluid floating layer, also fit to the observed compact intermetallic layers and scattered granular phase in this study. The combination of them finally formed the firm bond upon cooling. The dimensions and morphology of this region, also called the solder-bonding region (SBR) [15], determines the strength of the bond and thus the extent of die soldering. The SBR can be altered by various factors, in particular by the alloy composition, which is in fact the focus of this study. One of the most common methods is the addition of Mn, which is popular for its positive effects against die soldering. Although various publications mentioned that Mn affected the interface and improved demolding, the exact mechanism is still disputed [5,6,7,9,27]. Therefore, the following sections aim to analyze the influence of not only Mn, but also Mo, Co, and Cr on the intermetallic interface in detail.

### 3.2. Influence of Mn

**Figure 7:**
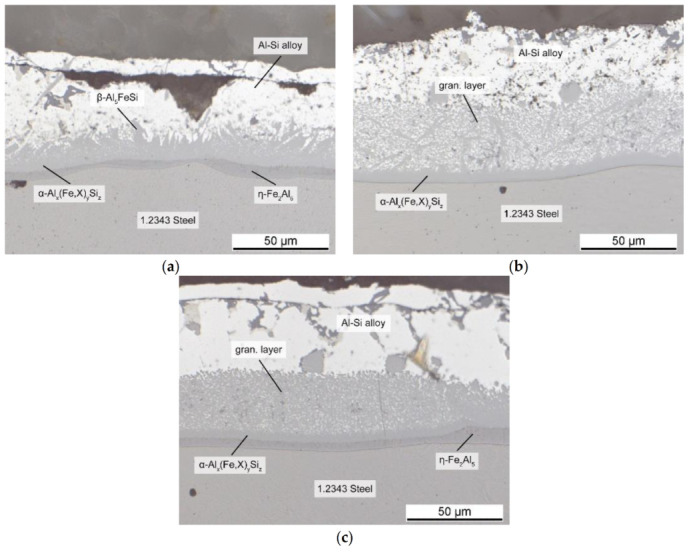
Intermetallic layers at the interface between 1.2343 tool steel and AlSi-alloy after the addition of (**a**) 0.2 Mn; (**b**) 0.4 Mn; (**c**) 0.8 Mn.

To understand the influence of the alloy composition on die soldering, we conducted immersion tests with different additions to the AlSi-alloy, as illustrated in Table 2. Figure 7 shows the interface for additions up to 0.8 wt.% Mn, an element commonly known for its positive effect against die soldering. The upper limit of the alloying addition was based on established practical values from the industry. The shape of the interface changed significantly when adding Mn; its overall thickness grew with increasing Mn content. Small additions led to a reduction in the length of the needle-shaped β-phase. The scattered granular phase on the other side became the main part of the boundary layer, and accounted for nearly 80% of its width. The interface with the AlSi-alloy became smooth and even, in direct contrast to the irregular course of the alloy without Mn in Figure 6.

**Figure 8:**
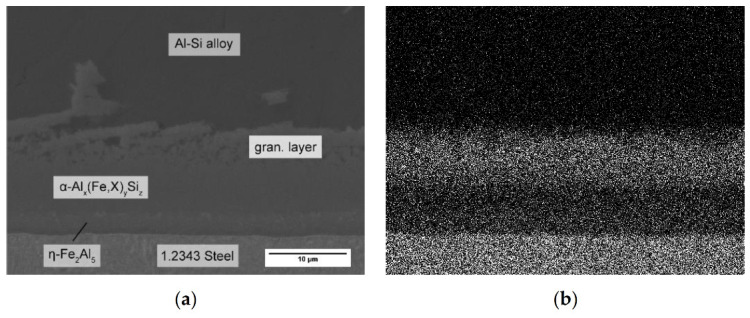
EDS mapping of the intermetallic interface between 1.2343 tool steel and AlSi-alloy with 0.8 wt.% Mn: (**a**) electron image; (**b**) Mn Ka1.

Although Mn is known for its positive effects against die soldering, there is little research about its effect mechanism at the interface. The usual purpose of alloying manganese is to prevent the formation of brittle β-Al_5_FeSi phase in the alloy and thus improve ductility. The suppression of the β-Al_5_FeSi phase by Mn is already well known and noted in many publications [28,29,30,31,32,33]. By occupying lattice sites of iron, it stabilizes the α-Al_15_(Fe,X)_3_Si_2_ phase. Unlike the monocline β-phase, it can take many shapes, with the most common morphology often described as Chinese-script [33]. Our results indicated that the effect of Mn on the intermetallic interface was based on the same mode of action. As mentioned before, both regions, the β-Al_5_FeSi phase and the surrounding AlSi-alloy, must be present to build a metallurgical bond. Figure 8 shows an EDS mapping of Mn at the intermetallic interface, which is mainly concentrated in the granular particles adjacent to the Al-alloy. The ternary layers at the interface can be divided into two compact phases and the granular region. The compact layers are the ternary α_H_-Al_12_Fe_3_Si_2_ phase, which were already present in the alloy without Mn, and the α_C_-Al_15_(Fe,X)_3_Si_2_ phase, which replaced the β-Al_5_FeSi phase and now represents the transition to the granular region, the intermetallic fraction of which also consists of it.

Thus, the stabilization of the α-phase led to a uniform layer without the needle-shaped surface responsible for interlocking of the two materials. As a secondary effect, however, the granular region, which consisted of fcc-Al and the α_C_-Al_15_(Fe,X)_3_Si_2_ phase, became very large when adding 0.4 wt.% Mn. The volume fraction of the fcc-Al phase in the granular layer was high compared to the intermetallic particles. It is yet to be examined whether these particles stay at the interface in an HPDC process due to the high forces and injection speed. However, if they form the transition from the compact intermetallic layer to the AlSi-alloy, the shape of the granular region could be of paramount importance to prevent die soldering. The fcc-Al fraction of the granular region should have at most the melting temperature of pure Al, which is still lower than the casting temperature. Therefore, the high volume fraction of fcc-Al in the granular region makes it likely for this area to become liquid in every casting cycle, thus offering the possibility for a repeated metallurgical bond forming. With higher Mn contents up to 0.8 wt.%, the thickness of the intermetallic interface grew just slightly more, but the volume fraction of the α_C_-Al_15_(Fe,X)_3_Si_2_ particles in the granular region became more dominant, while the fcc-Al fraction became significantly lower. This resulted in a smaller remelting area and therefore a lower liquid content at the interface in each casting cycle, reducing the tendency for die soldering. On the other hand, the increasing size of the granular region when adding Mn could also contribute to material loss, should the melt movement in the HPDC process drag the intermetallic particles away from the interface into the AlSi-alloy.

Overall, Mn caused a different morphology of the interface and thus prevented die soldering. Rather small additions decreased the size and number of the needle-like β-Al_5_FeSi phase. As mentioned above, this mode of action was similar to the effect of Mn on the iron rich phases in the alloy. However, as the iron content at the interface was much higher than in the alloy, the amount of Mn needed to suppress the β-phase should have been considerably higher. At 710 °C, more than 1 at % Fe was soluble in Al [22]. Considering an established ratio for Fe:Mn of about 1:1 for HPDC [28,34], this went well with the amount of Mn necessary to suppress die soldering in our study. The large granular region, with its high volume fraction of fcc-Al, could still result in partial remelting of the interface in every casting cycle, and thus die-soldering risk, which was due to eased bonding, increased rapidly. The obtained results suggest that the addition of up to 0.8 wt.% Mn was necessary to fully prevent die soldering. Only such high amounts led to a uniform intermetallic layer with a smooth boundary to the Al-alloy and a low Al fraction in the granular region, and therefore a small remelting region. Shankar and Apelian [5] also recommended the addition of 0.8 wt.% Mn for alloys with low iron content, which corresponds with the mechanism of action observed in this study.

### 3.3. Alternatives to Mn

Based on the obtained results for Mn, alternative elements to avoid die soldering were investigated. While Mn provided a well-established solution, it required relatively high additions. This led to an increased presence of brittle phases in the alloy, which significantly reduced its ductility. An alternative method, in which less addition would be sufficient, could offer great potential. Therefore, this study considered elements with similar behavior, atomic radius, or crystal lattice like Mn. Molybdenum, cobalt, and chromium are also known to occupy iron-lattice sites and stabilize the α_C_-Al_15_(Fe,X)_3_Si_2_ phase [35,36,37,38]. Thus, immersion tests were carried out to investigate their effect on the intermetallic layer morphology at the interface and to compare them to Mn. EDS mappings confirmed that the examined elements under investigation, just like Mn, accumulated mainly in the compact and granular α_C_-Al_15_(Fe,X)_3_Si_2_ phase.

**Figure 9:**
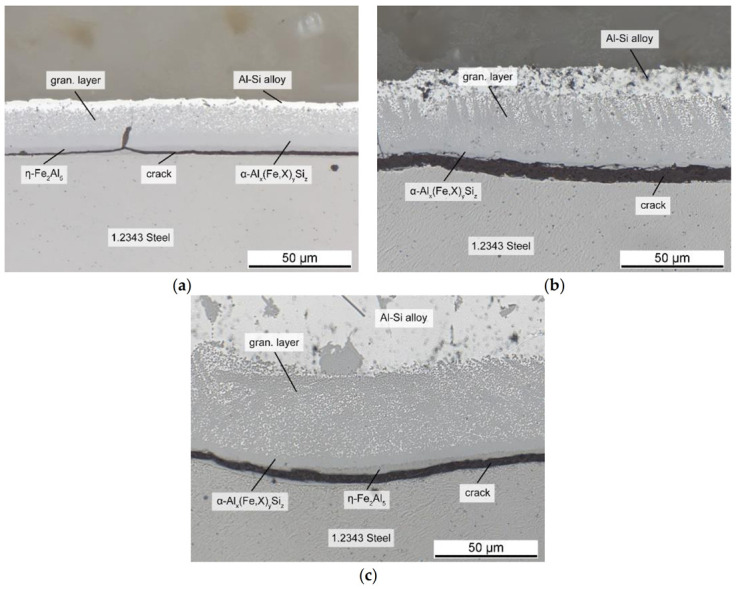
Intermetallic layers at the interface between 1.2343 tool steel and AlSi-alloy after the addition of (**a**) 0.1 Mo; (**b**) 0.2 Mo; (**c**) 0.3 Mo.

The morphology of the intermetallic interface changes was similar to Mn when using Mo or Co, though there were small differences, especially in the required content. As shown in Figure 9, Mo reduced the β-Al_5_FeSi phase and generated an extended granular region, like Mn. However, 0.3 wt.% Mo was as effective as 0.8 wt.% Mn. While the total layer thickness was even larger despite the lower content, both elements produced a similarly compact and smooth boundary to the Al-alloy, with a low fraction of fcc-Al phase in the granular layer. Incidentally, some cracks were visible that were related to preparation artefacts. Cobalt showed a similar behavior, with rising content up to 0.3 wt.%, but only minimal growth in layer thickness (not shown here). Although this behavior was desirable, the positive effect of Co did not act across the entire intermetallic interface, but only in sections. This probably was due to a stronger segregation tendency of Co in the Al-alloy. This behavior made it less efficient than Mn and Mo.

**Figure 10:**
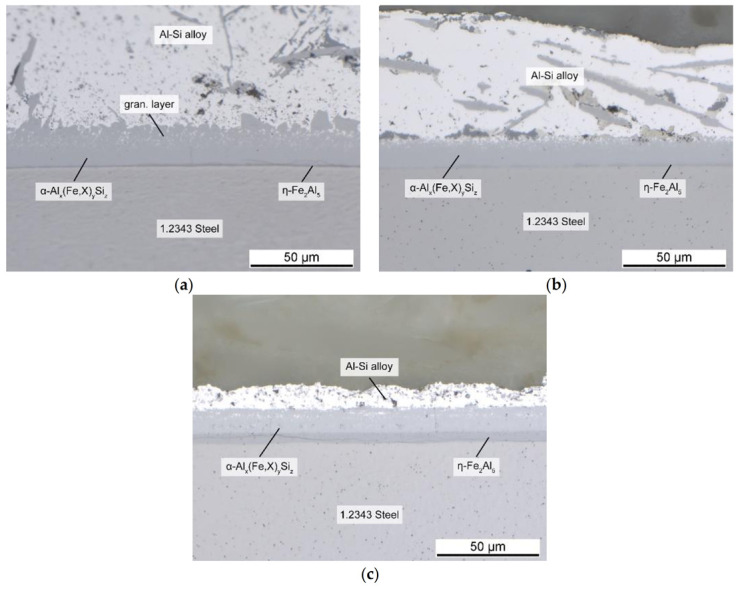
Intermetallic layers at the interface between 1.2343 tool steel and AlSi-alloy after the addition of (**a**) 0.1 Cr; (**b**) 0.2 Cr; (**c**) 0.3 Cr.

Just like the elements discussed above, chromium was mainly concentrated in the Al-rich area. It formed the α_C_-Al_15_(Fe,X)_3_Si_2_ phase, as shown by EDS measurements, and suppressed the needle-shaped β-Al_5_FeSi phase. However, contrary to the other elements, this did not lead to an increase in the intermetallic layer thickness. Instead, the interface became thinner with rising Cr. With an average of 17.5 μm at 0.2 wt.% Cr, it was approximately half the thickness compared to the reference alloy and about 1/3 compared to 0.3 wt.% Mo. This created a very favorable shape, as seen in Figure 10. The intermetallic interface between the die steel and AlSi-alloy was small and compact, with a negligible small granular region. No joint was formed because of the uniform course of the boundary layer. The very narrow range of the granular phase led to a very small remelting region within the intermetallic interface. This means that the interface remained almost completely solid for each casting cycle. Therefore, it should have acted like a protective layer on the die and hindered diffusion. Moreover, it reduced material loss of tool steel into the melt.

With more than 3 at % (see Table 3), Cr had the highest concentration of all elements in the ternary intermetallic layer. This should have resulted in a rising thickness of the respective phase. However, with 1.7 at %, it also occurred in a significantly higher amount in the binary η-Fe_2_Al_5_ layer than the other investigated elements, like Mn with 0.13 at % or Mo with 0.15 at %. This observation was also made by Shankar and Apelian [6], who also reported a reduced boundary layer thickness of about 60% caused by Cr addition [5]. Moreover, optical microscope images showed a broader η-Fe_2_Al_5_ layer for all Cr samples. While the thickness of the interface decreased overall, the η-Fe_2_Al_5_ layer showed the opposite behavior. Since this was the first phase to be formed [7], its increased thickness was likely to impede the diffusion of Fe atoms out of the die and thus the growth of the remaining intermetallic layers. However, the higher Cr content in the η-Fe_2_Al_5_ layer could also root in the diffusion of Cr out of the tool steel, which had about 5 wt.% Cr. This is supported by the fact, that the Cr content in the η-Fe_2_Al_5_ layer did not increase when compared to the reference alloy. Thus, the reason for the reduced thickness of the intermetallic interface when adding Cr to the alloy should be part of further research.

However, including the fact that 0.2 wt.% Cr is already sufficient to achieve these effects, it represents the most favorable way to prevent die soldering. Other publications like Mahta et al. [39] also reported that Cr required the lowest addition to avoid the formation of the β-Al_5_FeSi phase. It is an efficient alternative for Mn, and may improve the mechanical properties of the alloy due to the formation of Cr-bearing dispersoids [40]. Furthermore, because of its effectiveness compared to Mn, less Cr addition is required to prevent die soldering, which should result in less-brittle phases in the material and higher ductility.

**Figure 11:**
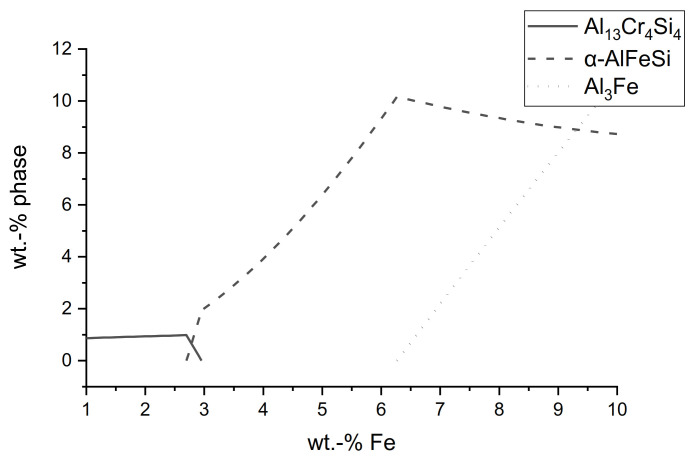
Thermodynamic simulation of the phase content over the iron content in AlSi_7_Cr_1_ at 700 °C using JMatPro (v10.2, Sente Software Ltd., Guildford, UK).

It is also worth noticing that the ternary AlCrSi- and AlMoSi-phase, which could be observed in the microstructure of components cast out of the AlSi-alloy as shown in Table 1 and Table 2 with Cr and Mo addition, did not occur at the steel interface. This is probably due to the higher iron content at the interface, rooting in the diffusion from the steel, compared to the small amount in the alloy itself. Figure 11 shows the simulated phase content over the iron content at a constant temperature of 700 °C for the reference alloy from Table 1 with the highest Cr addition from Table 2. The Cr content was raised from 0.3 wt.% to 1 wt.% for the simulation because of the additional Cr diffusing out of the tool steel. The temperature was set slightly lower than the pouring temperature to compensate for the heat transfer into the steel at the interface. At smaller Fe contents, like the 0.1 wt.% in the alloy, the ternary Al_13_Cr_4_Si_4_ phase was the only primary phase in equilibrium. At higher Fe contents, the Al_13_Cr_4_Si_4_ phase disappeared and the α_C_-Al_15_(Fe,X)_3_Si_2_ phase became dominant. Scheil solidification simulations confirmed the same trend for nonequilibrium transformations. This corresponded well with the different observations made at the interface and the alloy microstructure.

### 3.4. The Optimal Intermetallic Interface

**Figure 12:**
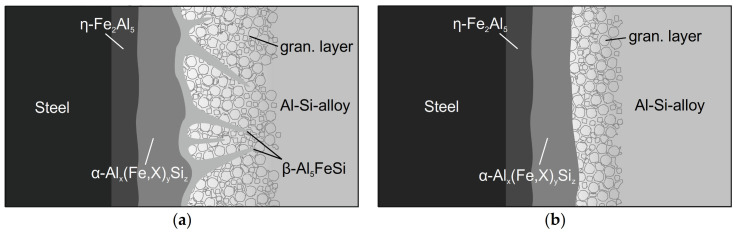
Schematic illustration of the intermetallic interface: (**a**) die soldering occurs; (**b**) no die soldering—optimal interface.

Based on the results of this study on the mechanism of die soldering and the influence of the alloying elements, an optimal composition and shape of the interface between an AlSi-alloy and hot work steel can be derived. Without preventive measures, the interface consisted of four different layers, which are illustrated in Figure 12a. Starting from the steel, the η-Fe_2_Al_5_ layer was the first one to form, followed by a ternary α_H_-Al_12_Fe_3_Si_2_ layer. Both layers ran evenly and did not contribute to die soldering. The combination of a β-Al_5_FeSi layer surrounded by a granular region and the AlSi-alloy was responsible for the bond between the two components is. The monocline β-phase grew into the aluminum alloy in a needle-shaped morphology. This led to an irregular and vast surface between the two materials, creating a joint. Furthermore, the fcc-Al phase fraction of the granular region and the AlSi-alloy adjacent to it had solidus temperatures lower than the casting temperature of the melt. While the β-phase remained solid, the surrounding region became liquid in every casting cycle and created new bonds. This resulted in severe die soldering and damage to the castings and the mold.

The shape of the intermetallic interface can be changed to a more favorable form by alloy optimization. By impeding the formation of the β-Al_5_FeSi phase and promoting the α_C_-Al_15_(Fe,X)_3_Si_2_ phase, the boundary changed from an irregular and needle-shaped form to a smoothly and evenly shaped form. This prevented the bonding between the components. Moreover, it was also important to reduce the thickness of the granular region and thus the overall intermetallic interface. It narrowed the remelting area within the interface, which became liquid in every casting cycle and offered the possibility of bonding. The ideal interface, illustrated in Figure 12b, runs smoothly and stays solid on the die. This way it acts like a protective layer on the mold, hindering diffusion and therefore die soldering.

## 4. Summary and Conclusions

In this study, immersion tests with hot work steel and hypoeutectic AlSi-alloys with systematically varied alloying elements were carried out to investigate the mechanism of die soldering and the influence of selected elements on its prevention. The following conclusions could be drawn:

1. Die soldering occurs due to the formation of intermetallic layers between the tool steel and the Al-alloy. The combined presence of the intermetallic β-Al_5_FeSi phase and a remelting region surrounding it are responsible for bonding. The intermetallic β-phase develops an irregular, needle-shaped boundary. With a solidus temperature lower than the casting temperature of the melt, the fcc-Al phase fraction of the granular layer and the AlSi-alloy become liquid in every casting cycle and solidify around the β-needles. This leads to a strong joint, and thus die soldering takes place.
2. The shape of the intermetallic interface can be altered by the alloy composition. The optimal intermetallic interface is free of the β-Al_5_FeSi phase and has an evenly shaped boundary to the granular region or the AlSi-alloy. The smooth course facilitates demolding. Additionally the granular region should be as narrow as possible to prevent remelting. In the ideal case, the intermetallic layer should stay as a solid and compact layer on the tool steel, impeding diffusion and thus die soldering. Still, the exact mechanism and role of the granular region needs further research.
3. Manganese inhibits the formation of the β-Al_5_FeSi phase. However, it creates a vast granular region. With rising Mn content, the volume fraction of intermetallic phase in the granular layer increases, while the possible remelting fcc-Al phase fraction decreases. Both effects improve the die-soldering behavior, yet to achieve them additions up to 0.8 wt.% are necessary. This leads to a higher proportion of brittle phases in the Al-alloy, which may decrease ductility.
4. Molybdenum and cobalt achieve similar effects to Mn. However, smaller additions of 0.3 wt.% are sufficient, which are presumably less harmful to ductility. While Mo presents an efficient alternative for Mn, Co seems to work only partially, with unaffected areas in between. This makes it a less-attractive solution to prevent die soldering.
5. Chromium is the most efficient element against die soldering. It inhibits the formation of the β-Al_5_FeSi phase and reduces the overall thickness of the intermetallic interface. In addition, the granular layer becomes negligible small. Optical microscopy and EDS measurements indicate the promotion of the η-Fe_2_Al_5_ phase, which could impede diffusion in early states of intermetallic formation. Cr is the only element investigated, which provides both features of an optimal intermetallic interface. Addition of 0.2 wt.% of Cr, which is sufficient to achieve these effects, offers a great potential to improve the properties of AlSi-alloys over Mn. However, additional investigations must be carried out regarding the interaction between the elements of this study to derive the most efficient alloy composition for high ductility, as well as die-soldering prevention. Further studies should also focus on the reducing influence of chromium on the layer thickness, in contrast to Mn and Mo, despite having similar effects on the intermetallic phases at the interface.

## Figures and Tables

**Table 1:** Composition of the 1.2343 tool steel and the reference aluminum alloy used in this work measured by spark spectroscopy (wt.%).

Element	C	Cr	Si	Mo	V	Mn	Fe	Mg
1.2343	0.38	5.30	1.00	1.20	0.40	0.40	Rest	-
Al-alloy (reference)	-	-	7.28	-	-	0.01	0.13	0.32

**Table 2:** Alloying additions to investigate the influence of the alloy composition (wt.%).

Element Addition			
Mn	Mo	Co	Cr
0.2	0.1	0.1	0.1
0.4	0.2	0.2	0.2
0.8	0.3	0.3	0.3

**Table 3:** Selected EDS analysis (at %) of different intermetallic layers observed in immersion tests with the AlSi-alloy.

Sample	Phase	Al	Si	Fe	Mn	Cr	Mo
Reference	η-Fe_2_Al_5_	63.34	5.79	28.31	0.17	1.98 *	0.27
Reference	β-Al_5_FeSi	70.24	14.85	14.13	0.06	0.41 *	0.12
Reference	α_H_-Al_12_Fe_3_Si_2_	69.18	12.32	18.50	0.10	1.18 *	0.14
0.8 Mn	α_C_-Al_15_(Fe,X)_3_Si_2_	72.18	10.33	15.13	2.36	1.06 *	0.29
0.3 Cr	α_C_-Al_15_(Fe,X)_3_Si_2_	71.83	10.07	13.28	0.10	3.27	0.33

* From steel.

## Data Availability

The data presented in this study are available on request from the corresponding author. The data are not publicly available due to restrictions on the part of AUDI AG.
